# Ultraviolet radiation reshapes the metabolome of skin commensal bacteria, influencing AhR signaling and barrier function

**DOI:** 10.1128/aem.02385-25

**Published:** 2026-03-18

**Authors:** Steven D. Mercer, Abigail Elias, George Taylor, Geoff S. Briggs, Michael Bell, Andrew J. McBain, Catherine A. O'Neill

**Affiliations:** 1 Division of Musculoskeletal and Dermatological Sciences, University of Manchester 523371, Manchester, United Kingdom; 2 Biological Mass Spectrometry Core Research Facility, University of Manchester 5292, Manchester, United Kingdom; 3 No.7 Beauty Company, The Boots Group 632395 , Nottingham, United Kingdom; 4 Division of Pharmacy and Optometry, University of Manchester 14301, Manchester, United Kingdom; University of Illinois Urbana-Champaign, Urbana, Illinois, USA

**Keywords:** microbiome, metabolites, tryptophan, aryl hydrocarbon receptor, ultraviolet radiation, skin, barrier function

## Abstract

Ultraviolet radiation (UVR) is an environmental stressor to the skin and its associated microbiota. Many commensal bacteria produce tryptophan-derived metabolites that modulate epidermal barrier function through aryl hydrocarbon receptor (AhR) signaling. However, the effects of UVR on these metabolic outputs and the subsequent effects on skin barrier function remain unclear. This study examined these interactions. Individual skin commensal bacteria were irradiated with 37.5 mJ/cm² or 150 mJ/cm² of solar-simulated radiation (SSR). Cell-free supernatants (CFSNs) were collected and analyzed for tryptophan metabolites (using LC–MS), untargeted metabolites (using GC–MS), and AhR agonist activity (via a reporter assay). The effects on barrier function were assessed by measuring transepithelial electrical resistance (TEER) of CFSN-treated keratinocyte cultures. Time-course expression of tryptophan-related genes after irradiation was measured by qPCR. Unirradiated bacterial metabolomes were species-specific. Following irradiation, the abundance of metabolites generated by the indole pathway generally increased after 37.5 mJ but decreased following 150 mJ. Untargeted analyses revealed several decreases in amino and organic acid production after high-dose SSR, while 37.5 mJ resulted in fewer changes. Low-dose SSR upregulated genes involved in tryptophan metabolism (*ipdC*, *ALDH*) and synthesis (*trpE*). AhR agonism increased in 8/11 organisms following irradiation, statistically correlating with increased levels of indole-pathway metabolites (indole-3-acetamide, tryptophol, indole-3-carboxaldehyde, and tryptamine). Keratinocytes treated with irradiated *Staphylococcus hominis*, *Micrococcus luteus*, and *Staphylococcus capitis* CFSNs showed enhanced TEER, concurrent with increased AhR activation; inhibition of the AhR removed this effect. UVR significantly alters the metabolomes of skin commensal bacteria, with knock-on effects for AhR signaling and barrier integrity, potentially influencing the skin’s response to UVR.

**IMPORTANCE:**

The skin and its commensal bacteria are regularly exposed to ultraviolet radiation (UVR). Many skin bacteria generate tryptophan-derived metabolites that influence host physiology through activation of the aryl hydrocarbon receptor (AhR), a key regulator of barrier integrity and stress responses. However, the impact of UVR on the metabolic activity of skin commensals and the downstream consequences for epidermal barrier function remains poorly understood. Here, we show that UVR significantly alters the metabolic outputs of diverse skin commensals, shifting production of indole-pathway metabolites and modulating AhR agonist activity. These changes translated into measurable effects on keratinocyte barrier function, including enhanced transepithelial electrical resistance following UVR exposure. Our findings reveal a previously underappreciated role for bacterial photometabolism in shaping epidermal barrier regulation and host responses to UVR.

## INTRODUCTION

The skin provides a physical and immunological barrier that protects the host from environmental insults, pathogens, and dehydration. Increasingly, it is clear that the skin does not perform these functions in isolation. Growing recognition for the role of the skin microbiome in epidermal health has led to several reports that the microbiome is essential for barrier development, maintenance, immune response, and protection from external stimuli such as ultraviolet radiation (UVR) (1–3). The barrier-modulating properties of the microbiome are now known to be mediated, in part, through the production of microbial metabolites interacting with the human aryl hydrocarbon receptor (AhR) (1). However, the role of UVR in this interplay has only recently been hypothesized (4).

Skin microbiome-derived metabolites, particularly indoles from tryptophan, activate the AhR (5). This transcription factor, present on numerous cell types, influences several genes relating to skin health, including differentiation, immune function, detoxification, and barrier function (6). Studies in models of atopic dermatitis have shown that AhR ligands can restore barrier function by increasing the expression of filaggrin, loricrin, and tight junction proteins (7–9). Additionally, Uberoi et al. (1) reported that germ-free (GF) mice had significantly increased transepidermal water loss, and keratinocytes derived from these mice had decreased barrier integrity (measured by transepithelial electrical resistance [TEER]) compared to specific pathogen-free mice. Importantly, the addition of AhR ligands or an artificial consortium of human skin commensal bacteria was able to restore barrier function, even in tape-stripped skin. A later study by Uberoi et al. (10) reported that, as well as impaired barrier function, GF mice had reduced abundance of some tryptophan metabolites on the skin and that addition of these metabolites restored barrier integrity through AhR signaling.

UVR is a significant stimulus to the skin, with excess exposure leading to increased barrier permeability (among other responses) (11). There is also evidence that UVR can alter the composition and abundance of the microbiome (12–14). Furthermore, the microbiome strongly influences the skin’s response to UVR. For example, Patra et al. (3) reported that germ-free (GF) mice show greater dermal mast cell and macrophage infiltration than wild-type (WT) animals, whereas WT mice exhibit more epidermal hyperplasia and neutrophil influx than their GF counterparts. In addition, the presence of a skin microbiome reportedly enhanced UV-induced keratinocyte proliferation and accelerated epidermal barrier repair, suggesting that commensal microbes modulate both immune and barrier responses following UV exposure (3). In another study, it was reported that eDNA from *Staphylococcal* biofilm protected keratinocytes from UVB-induced apoptosis (15), while butyrate produced from *Staphylococcus epidermidis* can suppress IL-6 and inflammation in mice following UVB exposure (16).

Mechanisms linking the microbiome to UVR responses remain incompletely defined. However, we propose that some of these effects may be mediated through changes in the production of microbial metabolites, leading to altered AhR signaling. Examples in skin are limited, but community studies suggest that UVR can drive metabolic reprogramming. For example, in wastewater, UVR reduces microbial diversity, suppresses amino acid, lipid, terpenoid, and polyketide pathways, and progressively downregulates nitrogen metabolism genes (17). Furthermore, as the microbial metabolome is highly dependent on the composition of the microbiome (18, 19), changes in bacterial species and abundance following UVR exposure could also alter the metabolome (4).

Here, we consider this hypothesis by assessing how solar-simulated radiation (SSR) reshapes the metabolic output of representative skin commensals and by determining the downstream consequences for AhR signaling and epidermal barrier integrity.

## MATERIALS AND METHODS

### Isolation and culture of skin commensal bacteria

A biobank of bacteria from the skin microbiome was collected as previously described (5). Briefly, swabs were collected from the scalp, forehead, volar forearm, and toe web of five healthy volunteers. All research was performed in accordance with relevant guidelines/regulations, and informed consent was obtained from all participants.Samples were cultured on various media to isolate specific bacterial groups: tryptic soy agar (Sigma-Aldrich, Germany) with 5% sheep’s blood (VWR, UK) for aerobic bacteria, mannitol salt agar for mannitol-fermenting *Staphylococci*, MacConkey agar for gram-negative bacteria, Wilkins-Chalgren agar for anaerobic bacteria, Lactobacilli MRS agar for *Lactobacilli*, and nitrofuran-containing agar for *Micrococcus* and *Corynebacterium*. Pure colonies were identified using 16S rRNA gene sequencing and stored in biobank beads at −80°C. The bacterial species included in this study are listed in Table 1, and the 16S rRNA accessions are shown in Table S1 .

**TABLE 1:** List of organisms used throughout the study

Organism	Abbreviation
*Staphylococcus aureus*	*S. aureus*
*Micrococcus luteus*	*M. luteus*
*Brachybacterium rhamnosum*	*B. rhamnosum*
*Kocuria rhizophila*	*K. rhizophila*
*Staphylococcus warneri*	*S. warneri*
*Kocuria marina*	*K. marina*
*Staphylococcus hominis*	*S. hominis*
*Staphylococcus capitis*	*S. capitis*
*Staphylococcus lugdunensis*	*S. lugdunensis*
*Staphylococcus epidermidis*	*S. epidermidis*
*Kocuria palustris*	*K. palustris*

### Preparation of cell-free supernatants

Tryptic soy broth (Sigma-Aldrich) was inoculated with overnight cultures and incubated at 37°C until the stationary phase was reached for all organisms (48 h). Cells were then resuspended in 200 µL of phosphate buffered saline to a final concentration of 1 × 10^7^ (*K. palustris* and *S. hominis* only) or 1 × 10^8^ CFU/mL. Cultures were irradiated with 0, 37.5, or 150 mJ/cm^2^ (mJ) of SSR using an Oriel SOL-UV-6 Solar Simulator (Newport Corporation, USA). Dosages of SSR were calculated using the following equations, where irradiance was determined by a UVX radiometer (Ultraviolet Products, UVP, Germany).


$$\begin{array}{l} True\;erythemally\text{-}weighted\;irradiance\;(True\;irradiance)=Radiometer\;value\times 3.52^{*}\times \;0.0212^{**} \end{array}\;\begin{array}{l} Exposure\;time\;(sec)=\frac{\text{Dose required }(mJ/{cm}^{2})}{\text{True irradiance }(mW/{cm}^{2})} \end{array}$$

where * represents externally validated calibration factor and ** represents adjustment value to derive the erythemally weighted irradiance based on the International Commission on Illumination (CIE) erythema action spectrum.

Following irradiation, bacteria were pelleted by centrifugation at 1,520 × *g* for 5 min and resuspended in custom RPMI-1640 (RPMI: RPMI-1640 with 2.0 g/L NaHCO_3_, without tryptophan, L-glutamine, and phenol red) supplemented with 0.05% tryptophan and 200 mM L-glutamine. Cultures were maintained in the dark for 5 days at 37°C to allow for metabolite accumulation, before the cell-free supernatants (CFSNs) were collected using 0.22 µm syringe filters (Starlab, UK) and stored at −20°C for downstream applications. The RPMI control used as a comparator in multiple experiments contained identical supplements and was subjected to the same incubation conditions as the experimental samples.

### Measurement of intracellular reactive oxygen species production

Reactive oxygen species (ROS) production following UVR irradiation was assessed using a DCFDA/H2DCFDA cellular ROS assay kit (Abcam, UK). Prior to irradiation, bacterial cultures were pelleted by centrifugation at 1,520 × *g* for 10 min before being washed and resuspended in 150 µL of Ringer’s solution at 1 × 10^7^ or 1 × 10^8^ CFU/mL. Dichlorofluorescin diacetate (DCF-DA; 30 µM) was then added to each well (50 µL), and plates were incubated for 30 min at 37°C. Following incubation, cultures were irradiated with 0 (control), 37.5, or 150 mJ/cm^2^ of SSR. Fluorescence was then measured 10 min after irradiation using a CLARIOstar microplate reader (BMG Labtech, Germany) (excitation: 485 nm; emission: 535 nm). Mean fluorescence of blank wells containing 150 µL of Ringer’s solution and 50 µL of 30 µM DCF-DA was deducted from individual sample values.

### LC–MS and GC–MS of metabolites

All samples and standards were spiked with ^13^C_6_-5-HIAA (100 ng/mL). External calibration (1–500 ng/mL) covered 14 tryptophan-related analytes (listed in Table S1). Blanks and pooled QCs were included in each batch; calibrations required *R*² ≥ 0.99.

### Targeted LC–MS (tryptophan metabolites)

Analyses were performed on a SCIEX ExionLC coupled to a SCIEX 7600 ZenoTOF Q-TOF (positive ESI). Ten-microliter injections were separated on a Thermo Accucore C18 (150 × 2.1 mm, 2.6 µm) using water/0.1% formic acid (A) and acetonitrile/0.1% formic acid (B) at 0.3 mL/min: 5% B (1 min) to 100% B (7 min), hold for 2 min, then re-equilibrate for 4 min (total 15 min/sample). Data were acquired by DIA/SWATH (*m*/*z* 50–1,000) with variable windows. Collision energy followed CE = 0.084 × *m*/*z* + 12 V (capped at 55 V). Data processing used PeakView 2.2 and MultiQuant 3.0.2.

### Untargeted GC–MS

Aliquots (100 µL) were dried, methoximated (50 µL methoxamine HCl in pyridine, 60°C, 15 min), and then silylated (50 µL BSTFA + 1% TMCS, 60°C, 15 min). Samples were analyzed on an Agilent Intuvo 9000 GC with DB5-MS, 30 m × 250 µm × 2.5 µm, coupled to a 5977B MSD. One microliter was injected (split 10:1, injector at 250°C) with helium carrier at ~1.1 mL/min. Oven: 60°C (1 min) to 325°C at 10°C/min, hold 10 min (total 37.5 min). MS settings: EI, *m*/*z* 50–600, solvent delay 5 min; transfer line/source/quadrupole 290/250/150°C. Data were processed in MassHunter (Qual v10, Unknowns v10.2, Quant v10.2).

### qPCR

Bacterial cells were cultured and irradiated as described above. RNA was extracted at 6, 16, 24, 72, and 120 h post-irradiation. Cells were lysed in 1 mL TRIzol (ThermoFisher Scientific) using bead-beating tubes for 5 min. Chloroform (200 µL; Sigma-Aldrich) was added, mixed for 15 s, and the mixture was centrifuged (12,000 × *g*, 4°C, 15 min). The aqueous phase was purified using the RNeasy Mini Kit (Qiagen, Netherlands), quantified with the Qubit RNA BR Assay (ThermoFisher Scientific), and stored at −80°C.

Prior to analysis, RNA samples were treated with DNase I (Promega, USA) for 30 min at 37°C, followed by heat inactivation at 65°C for 10 min in stop solution. Quantitative PCR was performed using the TaqMan RNA-to-CT Kit (ThermoFisher Scientific) with custom TaqMan gene expression assays (catalog: 4331348), using 2 ng RNA per reaction. Reactions were run on a QuantStudio 12K Flex Real-Time PCR System under the following conditions: 48°C for 15 min; 95°C for 10 min; and 40 cycles of 95°C for 15 s and 60°C for 1 min (data acquisition at 60°C). Gene expression was analyzed using the comparative CT (2⁻^ΔΔCT^) method, with normalization to the housekeeping gene *rpoB*.

Custom TaqMan gene expression assays were designed by ThermoFisher Scientific using representative nucleotide sequences. Target gene sequences were identified through searches of the NCBI database and used as templates for assay design. Gene identity was confirmed by BLASTn and/or BLASTx analysis against *Staphylococcus hominis* reference genomes and the NCBI RefSeq protein database. ThermoFisher custom assay IDs, NCBI/Blast accession numbers used for assay design, and primer and probe sequences are provided in Table S2. No-template and no-reverse-transcription controls were included in all runs to confirm the absence of contamination or genomic DNA amplification.

### AhR luciferase reporter assay

The Indigo Biosciences Human AhR Reporter Assay System was used according to the manufacturer’s instructions. Briefly, luciferase reporter cells were recovered from −80°C storage and seeded into 96-well plates for 4 h. Culture medium (150 µL) was then added alongside CFSNs (50 µL) and incubated for 24 h (37°C, 5% CO_2_). Following incubation, treatment medium was discarded, and a luciferase detection reagent was added to each well. Luminescence was quantified using a CLARIOstar microplate reader, and AhR fold activation was calculated as sample luminescence/background luminescence.

### Culture of primary human keratinocytes

Juvenile normal human keratinocytes (NHEKs; catalog: C-12005) were cultured in KGM: basal keratinocyte medium with keratinocyte supplement mix and CaCl_2_, until 80%–90% confluency was reached. Trypan blue exclusion was used for viable counts to determine seeding densities. All cell culture incubations were performed at 37°C, 5% CO_2_. For storage, cells were resuspended in 90% fetal bovine serum (Sigma-Aldrich) and 10% DMSO and frozen in liquid nitrogen vapor.

### Assessment of keratinocyte viability following CFSN treatment

NHEKs were seeded into TC-treated 96-well plates (1 × 10^4^ cells/well) and cultured until 70%–80% confluency was reached. CFSNs (25 µL) were then added alongside KGM (75 µL) and incubated for 24 h. 3-(4,5-dimethylthiazol-2-yl)-2,5-diphenyltetrazolium bromide (10 µL per well, 5 mg/mL) was added to each well and incubated for 3 h. Liquid was removed, and 150 µL of DMSO was added. Plates were then shaken (300 RPM) for 10 min before absorbance readings were measured at 540 nm with a background reading at 570 nm using a CLARIOstar microplate reader. Percent viability was calculated as a percentage of the control (cells grown in culture media only). Each sample was run in duplicate.

### Transepithelial electrical resistance

NHEKs were seeded into 24-well transwells with 0.4 µm pores (Corning, UK) (2 × 10⁴ cells/well) using KGM and cultured until they reached 90% confluence. TEER measurements were recorded using a World Precision Instruments Evometer with a chopstick electrode (WPI, UK). The medium was then replaced with Epilife+: Epilife media supplemented with 1.8794 mM CaCl_2_ and 1% human keratinocyte growth supplement (Thermofisher Scientific). Fresh medium was added to both the apical (225 µL) and basal (800 µL) compartments. Cells were cultured for an additional 24 h before TEER measurements were taken again. CFSNs (75 µL) were then introduced into the apical compartment, and cells were cultured for a further 3 days, with TEER readings recorded every 24 h. Sample values were determined by subtracting the media-only readings from the sample readings, and fold changes were calculated by dividing sample values by the blank control (cells grown in culture media only).

For some conditions, 10 µM of the AhR antagonist CH-223191 (Sigma-Aldrich) was added 24 h post-calcium switching, 1 h before treatment with CFSNs.

### Statistical analysis

Analysis of the LC–MS, GC–MS, and AhR activation data was performed in R (v4.5.0). Data handling used tidyverse and readxl. MixOmics and ropls were used for multivariate analyses (partial least squares discriminant analysis [PLS-DA]); stats for ANOVA, Welch’s *t*-tests, and Spearman correlations; emmeans and multcomp for Tukey and Dunnett *post hoc* tests; vegan for Shannon diversity; pheatmap and ComplexHeatmap for heatmaps; ggplot2 for figures; and stats:p.adjust for Benjamini–Hochberg FDR correction. qPCR and TEER data were analyzed in GraphPad Prism (v10.6.0) using a Kruskal-Wallis test with a Dunn’s *post hoc* (qPCR), or a one-way ANOVA with a Tukey’s *post hoc* (TEER).

## RESULTS

### Skin commensal bacteria have distinct metabolomes

**Fig 1:**
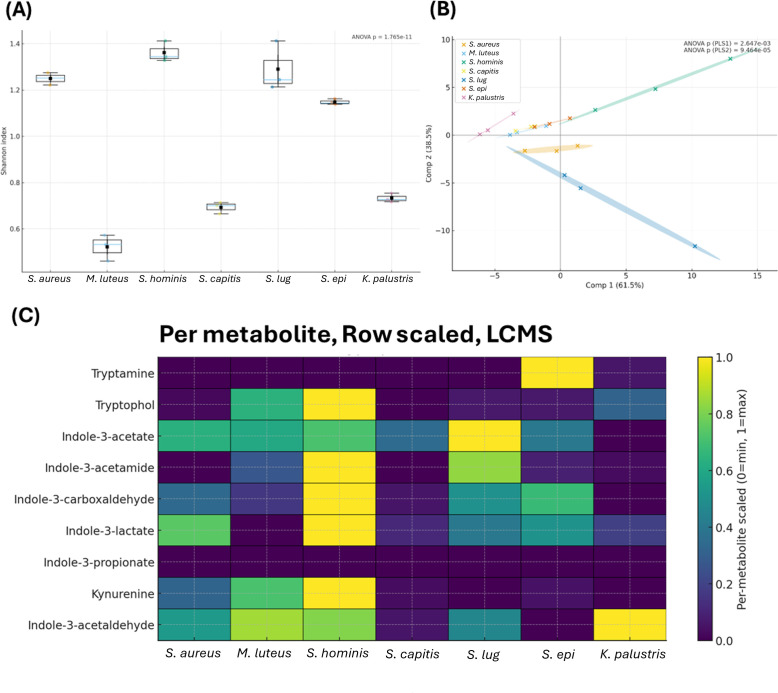
Metabolomic differences among seven unirradiated skin commensals. (**A**) Alpha diversity (Shannon index) across organisms; *P* value from one-way ANOVA. (**B**) Two-component partial least squares discriminant analysis (PLS-DA) of combined LC–MS/GC–MS data; axes show % variance and 68% confidence ellipses; *P* values from one-way ANOVA on PLS1/PLS2 scores. (**C**) LC–MS heatmap with per-metabolite (row) min–max scaling (0 = min, 1 = max). *S. lug*: *S. lugdunensis*, *S. epi*: *S. epidermidis.* Biological *n* = 3 per organism.

Skin commensal bacteria metabolize tryptophan, as well as other amino acids, into a diverse range of metabolites (5). However, comparative metabolomic profiles across skin-derived species are scarce. Therefore, we analyzed the metabolomes of seven unirradiated skin commensals (Fig. 1).

Metabolomes differed markedly across unirradiated organisms, consistent with previous findings (5). Combined analysis of targeted LC–MS (tryptophan pathway) and untargeted GC–MS showed organism-specific profiles. Shannon diversity was highest in *S. hominis*, followed by *S. aureus*, *S. lugdunensis*, and *S. epidermidis*; *M. luteus*, *S. capitis*, and *K. palustris* had the lowest diversity. Only the diversity of *S. aureus*, *S. hominis*, and *S. lugdunensis* did not significantly differ from each other (Fig. 1A).

A two-component PLS-DA resolved clear, organism-specific clustering (Fig. 1B), with scores differing by organism on both components (*P* < 0.0001). Loadings indicated that PLS1 was driven by indole-pathway metabolites (LC–MS: indole-3-acetamide, indole-3-carboxaldehyde, indole-3-lactate) plus amino acid/energy markers (GC–MS: L-leucine, trans-4-hydroxy-L-proline, 3-phenyllactic acid, L-ornithine, glycerol-phosphate, fumarate). PLS2 was dominated by aromatic/central-nitrogen metabolites (GC–MS: L-tyrosine, 2-hydroxybutyrate, 4-hydroxyphenylacetate, L-glutamine, 5,6-dihydro-5-methyluracil, L-proline, phosphoric acid, L-lysine) plus LC–MS indole-3-acetate. A per-metabolite (row-scaled) heatmap of tryptophan metabolites confirmed these patterns (Fig. 1C): *S. hominis* had the highest concentrations of indole-3-acetamide, indole-3-carboxaldehyde, indole-3-lactate, tryptophol, and kynurenine; *S. epidermidis* had high levels of tryptamine, while *S. lugdunensis* had elevated indole-3-acetate. *S. capitis* and *K. palustris* generally had the lowest metabolite production, except for indole-3-acetaldehyde, which was produced in high concentrations for *K. palustris*.

### Solar-simulated radiation alters the metabolome and gene expression of skin commensal bacteria

Next, we aimed to determine the effects of UVR on the metabolomes of skin commensals. Toward this, organisms were irradiated with 37.5 or 150 mJ of SSR; physiologically relevant doses corresponding to approximately 1 h 20 mins or 4 h of UVR exposure, respectively, on a day with a UV index of 6 (20). Both doses of SSR elicited a significant increase in intracellular reactive oxygen species (ROS) in 10 of the 11 strains tested, indicating that these irradiation parameters reliably induced a cellular redox response across the bacterial panel (Fig. S1).

**Fig 2:**
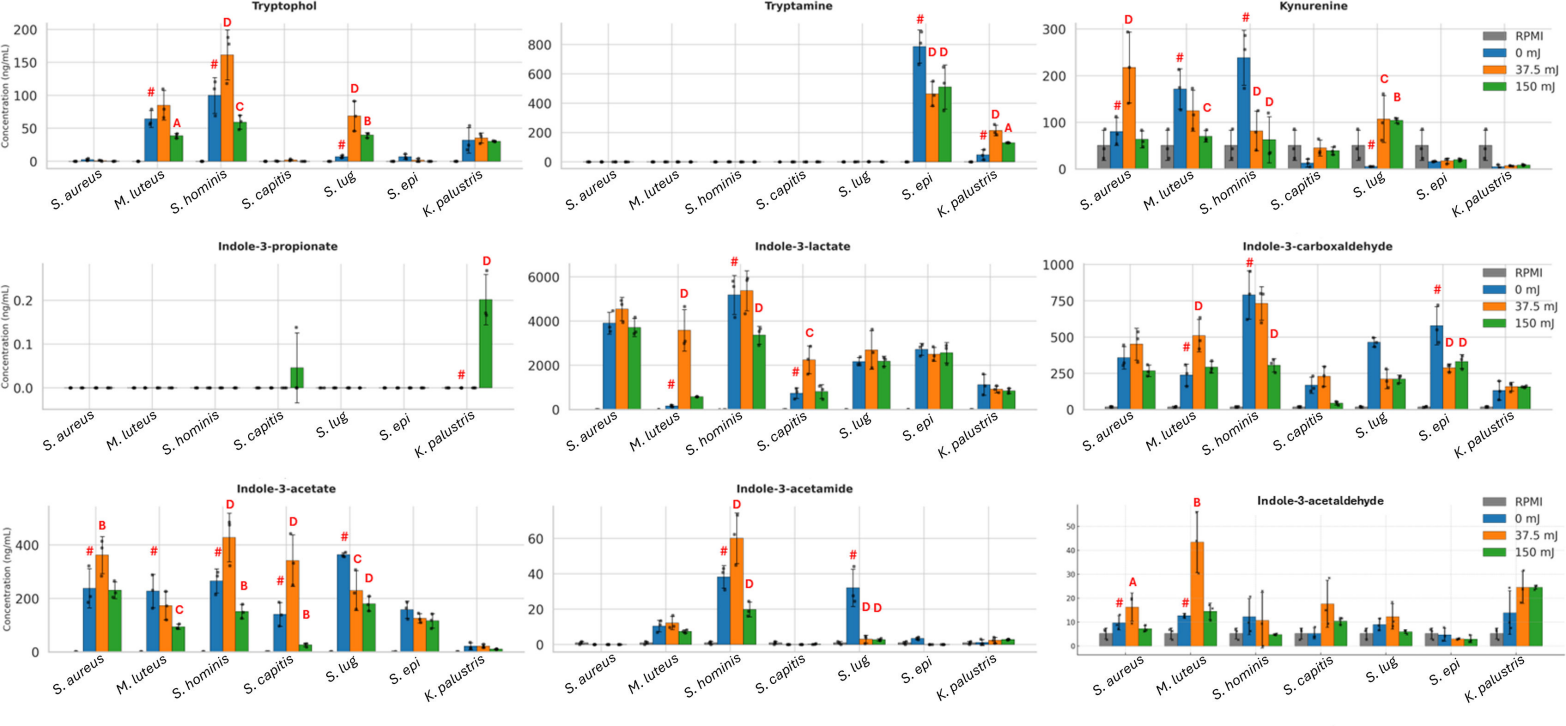
LC–MS of tryptophan metabolites for seven organisms before and after irradiation. Bars show the mean ± SD. Biological *n* = 3. *S. lug*: *S. lugdunensis*, *S. epi*: *S. epidermidis.* One-way ANOVA with a Dunnett’s *post hoc*. Statistics are compared to the 0 mJ CFSNs (#). A: *P* < 0.05, B: *P* < 0.01, C: *P* < 0.001, D: *P* < 0.0001.

The dose of SSR significantly altered the microbial metabolome, particularly regarding the production of tryptophan metabolites (Fig. 2). After exposure to 37.5 mJ of SSR, widespread, organism-specific enhancements in metabolite production were observed. In *S. hominis*, concentrations increased for indole-3-acetamide (1.57×, *P* < 0.0001), indole-3-acetate (1.61×, *P* < 0.0001), and tryptophol (1.62×, *P* < 0.0001). *M. luteus* showed some of the largest responses, including elevations in the production of indole-3-carboxaldehyde (2.14×, *P* < 0.0001), indole-3-acetaldehyde (3.42×, *P =* 0.0026), and indole-3-lactate (21.79×, *P* < 0.0001). In *S. aureus*, indole-3-acetate increased (1.52×, *P* = 0.0017), while *S. capitis* exhibited significant gains in indole-3-acetate (2.43×, *P* < 0.0001) and indole-3-lactate (3.03×, *P* = 0.0003). *S. lugdunensis* showed a marked rise in tryptophol (9.80×, *P* < 0.0001) and kynurenine (19.4×, *P =* 0.0008). Some decreases were seen following irradiation with 37.5 mJ. For example, *S. epidermidis* had reduced tryptamine and indole-3-carboxaldehyde (*P* < 0.0001), while *S. lugdunensis* had reduced indole-3-acetamide (*P* < 0.0001).

Irradiation with 150 mJ generally led to decreased metabolite production (Fig. 2). Significant reductions included *S. hominis*: indole-3-acetamide (0.52×, *P* < 0.0001) and tryptophol (0.59×, *P* = 0.0006); *M. luteus*: indole-3-acetate (0.41×, *P* = 0.0007), kynurenine (0.41×, *P* = 0.0009), and tryptophol (0.60×, *P* = 0.0390); *S. capitis*: indole-3-acetate (0.19×, *P* = 0.0041); *S. lugdunensis*: indole-3-acetamide (0.08×, *P* < 0.0001) and indole-3-acetate (0.50×, *P* < 0.0001); *S. epidermidis*: indole-3-carboxaldehyde (0.57×, *P* < 0.0001) and tryptamine (0.65×, *P* < 0.0001). Despite this, some increases were seen following irradiation with high-dose SSR. For example, *S. lugdunensis* showed a rise in kynurenine (18.9×, *P =* 0.0012).

The dose of SSR significantly altered microbial growth and viability (Fig. S2), and therefore, we considered that some of the observed decreases in metabolites were due to changes in microbial abundance, rather than altered microbial function. Area under the curve analysis of CFU counts performed at 0, 24, 48, 72, 96, and 120 h found that many organisms, including *B. rhamnosum*, *K. rhizophila*, *K. marina*, *S. capitis*, and *S. lugdunensis*, had reduced CFU/mL after irradiation with both doses. *S. aureus*, *S. lugdunensis*, and *S. epidermidis* had reduced CFU/mL only at 150 mJ. Only *M. luteus*, *S. warneri*, and *S. hominis* were unaffected by SSR. Interestingly, 37.5 mJ boosted bacterial growth of *S. aureus.* Notably, despite lower cell counts, some organisms (e.g., *S. epidermidis*) showed few reductions in production of tryptophan metabolites, implying that UVR may elevate per-cell metabolism, even where overall concentrations fall.

Irradiation appeared to trigger *de novo* synthesis of certain metabolites. No unirradiated organisms produced indole-3-propionate. However, following irradiation with 150 mJ of SSR, *S. capitis* and *K. palustris* both began production (*P* = 0.0101 and *P* < 0.0001, respectively), albeit at relatively low concentrations.

Certain organisms, particularly *K. palustris*, were extremely resilient to SSR and demonstrated limited changes in metabolite production. *S. epidermidis* also demonstrated few changes compared to other organisms. These were also the only organisms that produced tryptamine, which was decreased following irradiation for *S. epidermidis*, but increased for *K. palustris*.

Notably, tryptophan was highly abundant in the growth medium and remained above the limit of quantification for all organisms, indicating that observed differences in downstream metabolite profiles were not driven by substrate limitation.

**Fig 3:**
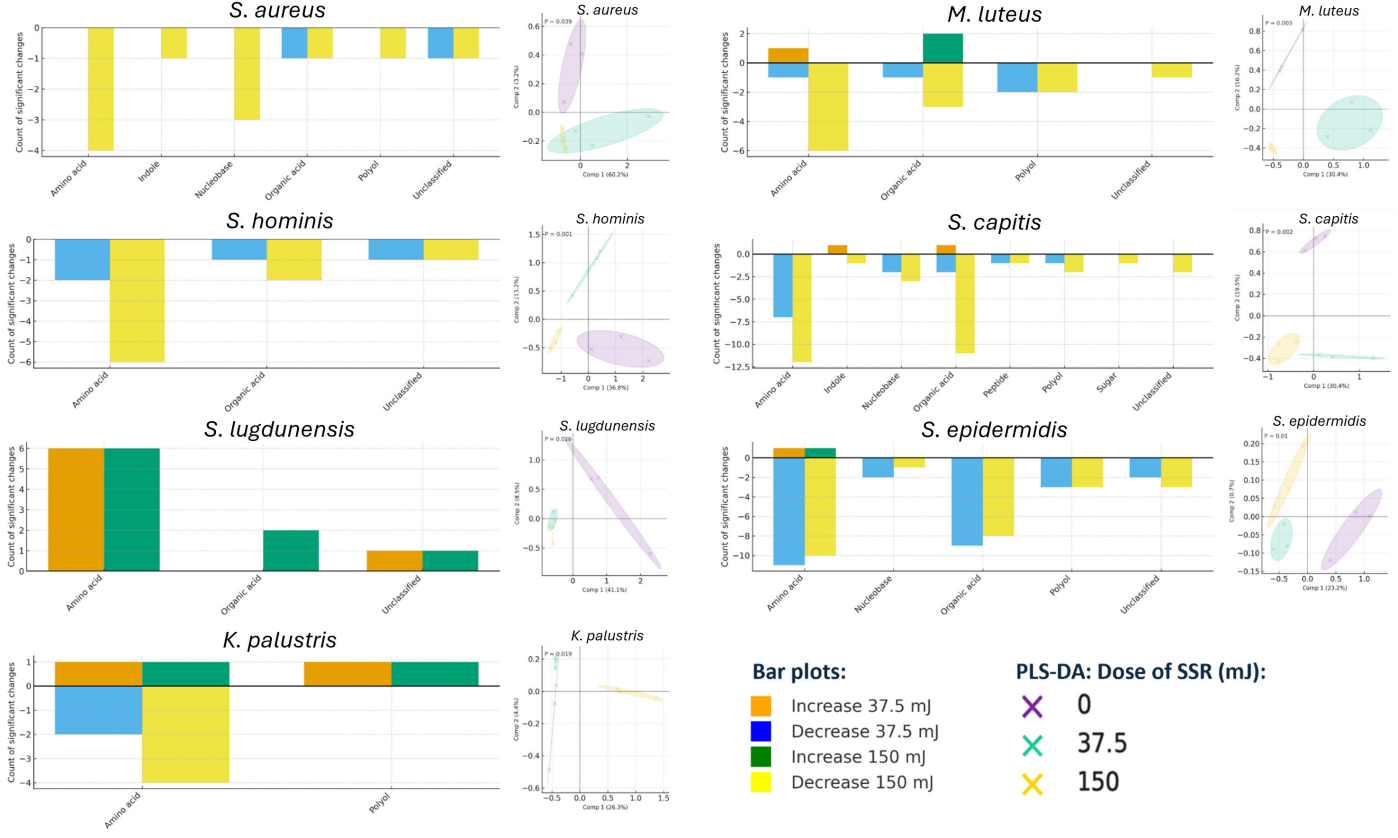
GC–MS of untargeted metabolites. For each organism, counts of metabolite groups (classified using PubChem identifiers) with significant changes are shown for 0 vs 37.5 mJ and 0 vs 150 mJ (average of *n* = 3 biological replicates). Significance was assessed with Welch’s *t*-test (*P* < 0.05). PLS-DA models are shown for each organism, axes show % variance and 68% confidence ellipses; group differences were evaluated by one-way ANOVA to obtain *P* values.

We next profiled the broader metabolome beyond tryptophan derivatives and detected 51 additional metabolites, many of which were primary metabolites (Fig. 3) (full data set available, https://doi.org/10.5281/zenodo.18349217). Irradiation elicited many significant changes with marked organism-to-organism variability, though all organisms showed distinct clustering based on the dose of SSR. *S. capitis* and *S. epidermidis* underwent the largest changes in metabolite profiles (PLS-DA, *P* = 0.002 and 0.01, respectively), characterized predominantly by decreases in metabolite abundance with similar responses across doses. This trend was seen for all organisms, with SSR significantly altering all PLS-DA clusters. Amino acids and organic acids were especially reduced following irradiation. Other species (*S. aureus*, *M. luteus*, and *S. hominis*) also exhibited declines in these classes, with a greater number of changes at 150 mJ. In contrast, *K. palustris* remained resilient, showing few alterations in primary metabolite secretion. Notably, *S. lugdunensis* displayed several increases and no decreases in primary metabolites, particularly within the amino acid group.

**Fig 4:**
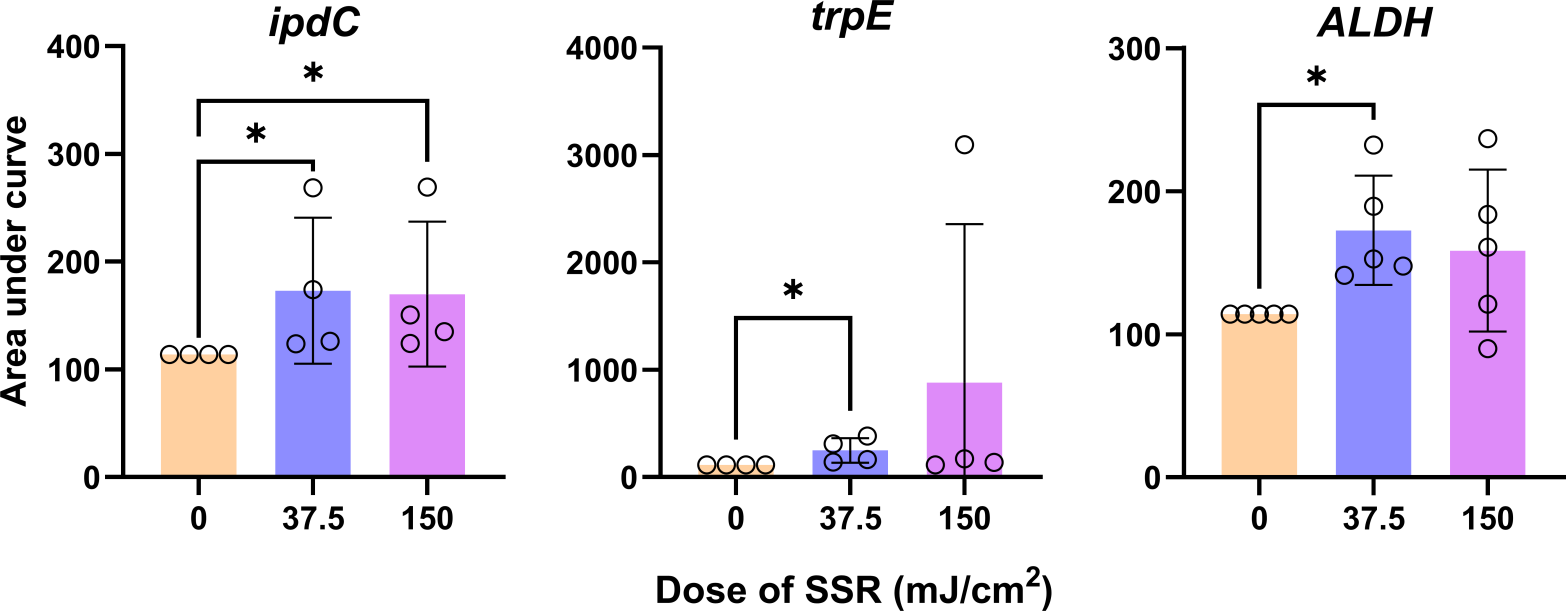
Expression of tryptophan synthesis/metabolism genes following irradiation with SSR. Area under the curve analysis was performed on 2^−ΔΔCT^ changes from a 120 h time course (6, 16, 24, 72, and 120 h). Indole-3-pyruvate decarboxylase (*ipdC*), anthranilate synthase component 1 (*trpE*), aldehyde dehydrogenase (*ALDH*). Biological *n* = 4−5, Kruskal-Wallis test with a Dunn’s *post hoc*: *P < 0.05.

We next aimed to determine how SSR alters bacterial gene expression. *S. hominis* was selected for this analysis as it exhibited several significant changes in tryptophan metabolite production in the LC–MS data set. Accordingly, a gene expression time course was performed on irradiated and unirradiated *S. hominis* for three genes relating to tryptophan synthesis (anthranilate synthase component 1: *trpE*) and metabolism (aldehyde dehydrogenase: *ALDH* and indole-3-pyruvate decarboxylase: *ipdC*). These data are shown as area under the curve (Fig. 4) and as mean values across the 120 h time course (Fig. S3). Concurrent with the increases in metabolite concentrations, *ipdC* significantly increased in expression following irradiation with both doses of SSR (37.5 mJ: *P* = 0.0434 and 150 mJ: *P* = 0.0252). Additionally, the expression of *ALDH* and *trpE* significantly increased after irradiation with 37.5 mJ of SSR (*P* = 0.0287 and 0.0189, respectively), while no change was seen following irradiation with 150 mJ.

### Altered metabolome leads to changes in AhR signaling

**Fig 5:**
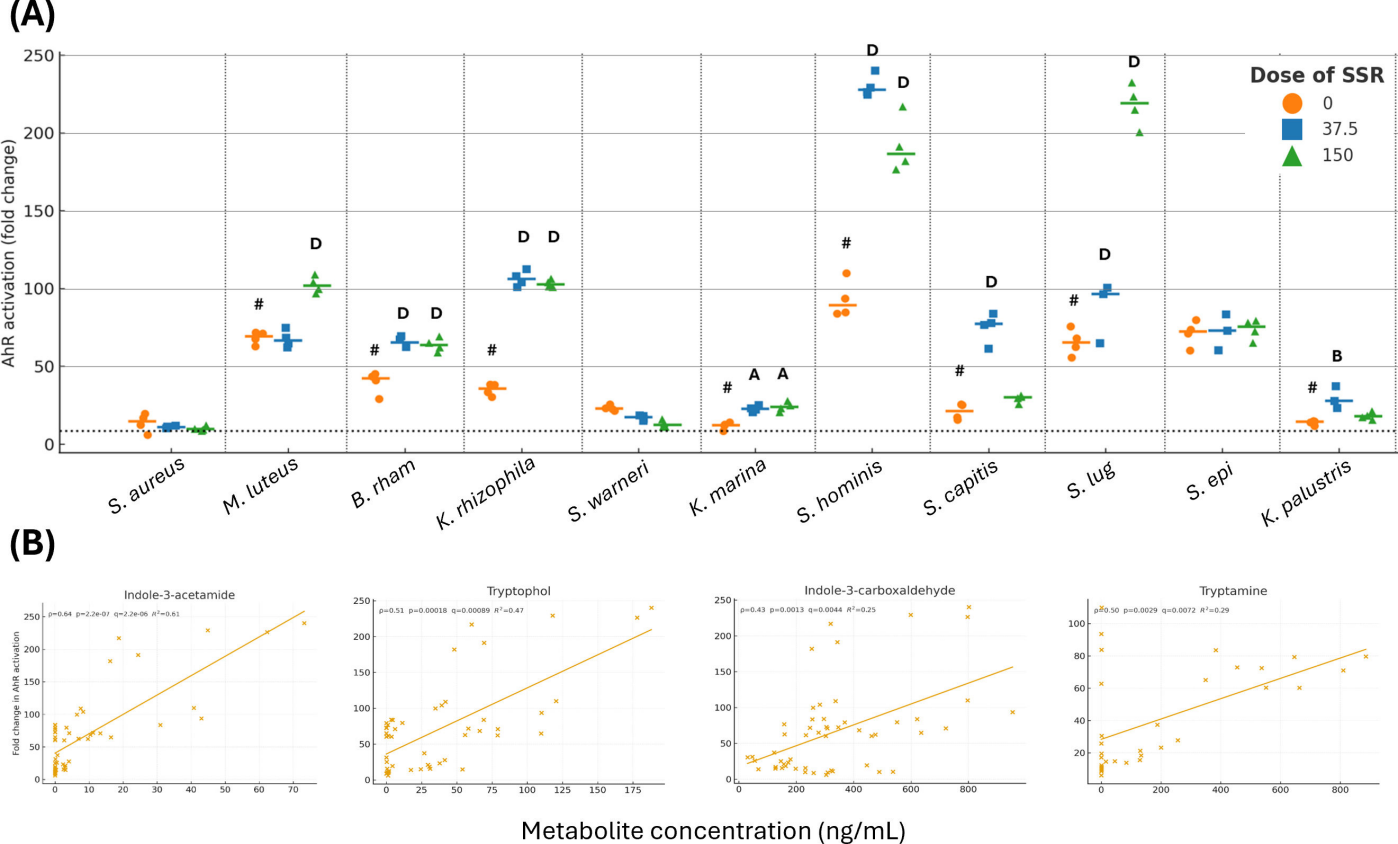
(**A**) Fold change in AhR activation after treatment with CFSNs from irradiated and unirradiated bacteria. The dotted line represents the mean fold change of the RPMI control. Biological *n* = 3–4. *B. rham*: *B. rhamnosum, S. lug*: *S. lugdunensis*, *S. epi*: *S. epidermidis.* Statistics are compared to the 0 mJ CFSNs (#) using a one-way ANOVA with a Dunnett’s *post hoc*. A: *P* < 0.05, B: *P* < 0.01, C: *P* < 0.001, D: *P* < 0.0001. (**B**) Spearman’s correlation of tryptophan metabolites and fold change in AhR activation. Only statistically significant metabolites are shown, which were determined by Benjamini–Hochberg FDR.

Due to the strong association between tryptophan metabolites and AhR signaling, we next assessed how altered metabolomes following irradiation affected AhR signaling (Fig. 5A). CFSNs from 11 irradiated and unirradiated bacteria were added to a luciferase reporter assay for 24 h, and the fold change in AhR activation was quantified.

For 8/11 organisms on the panel, CFSNs from unirradiated bacteria activated the AhR, with varying degrees of activation based on the organism. Following irradiation, CFSNs from 10/11 organisms activated the AhR for at least one dose of SSR: only *S. aureus* showed no activation for any condition. Many unirradiated *Kocuria* spp., as well as unirradiated *S. capitis*, had limited AhR activation but exhibited significant agonist activity after irradiation. Generally, *Staphylococci* species activated the AhR, and irradiation frequently augmented this effect. Many responses were dose-dependent: *M. luteus* increased activation only after irradiation with 150 mJ, whereas *S. capitis* increased activation following irradiation with 37.5 mJ. The CFSNs from *S. hominis*, which had high agonist activity when unirradiated, had increased potency at both doses of SSR, most notably at 37.5 mJ (*P* < 0.0001 for all comparisons). By contrast, CFSNs from irradiated *S. epidermidis* did not have altered AhR agonism for any dose of SSR.

Spearman’s correlation analysis (Fig. 5B) indicated that higher levels of indole-3-acetamide, tryptophol, indole-3-carboxaldehyde, and tryptamine were each associated with enhanced AhR activation (*P* < 0.0001, *P* = 0.00018, *P* = 0.0013, and *P* = 0.0029, respectively). No other metabolites significantly correlated with AhR activation.

Importantly, irradiation did not increase the toxicity of the CFSNs, as cell viability following 24 h of treatment was comparable to the unirradiated controls for all conditions (Fig. S4).

### CFSNs from irradiated bacteria enhance barrier integrity

**Fig 6:**
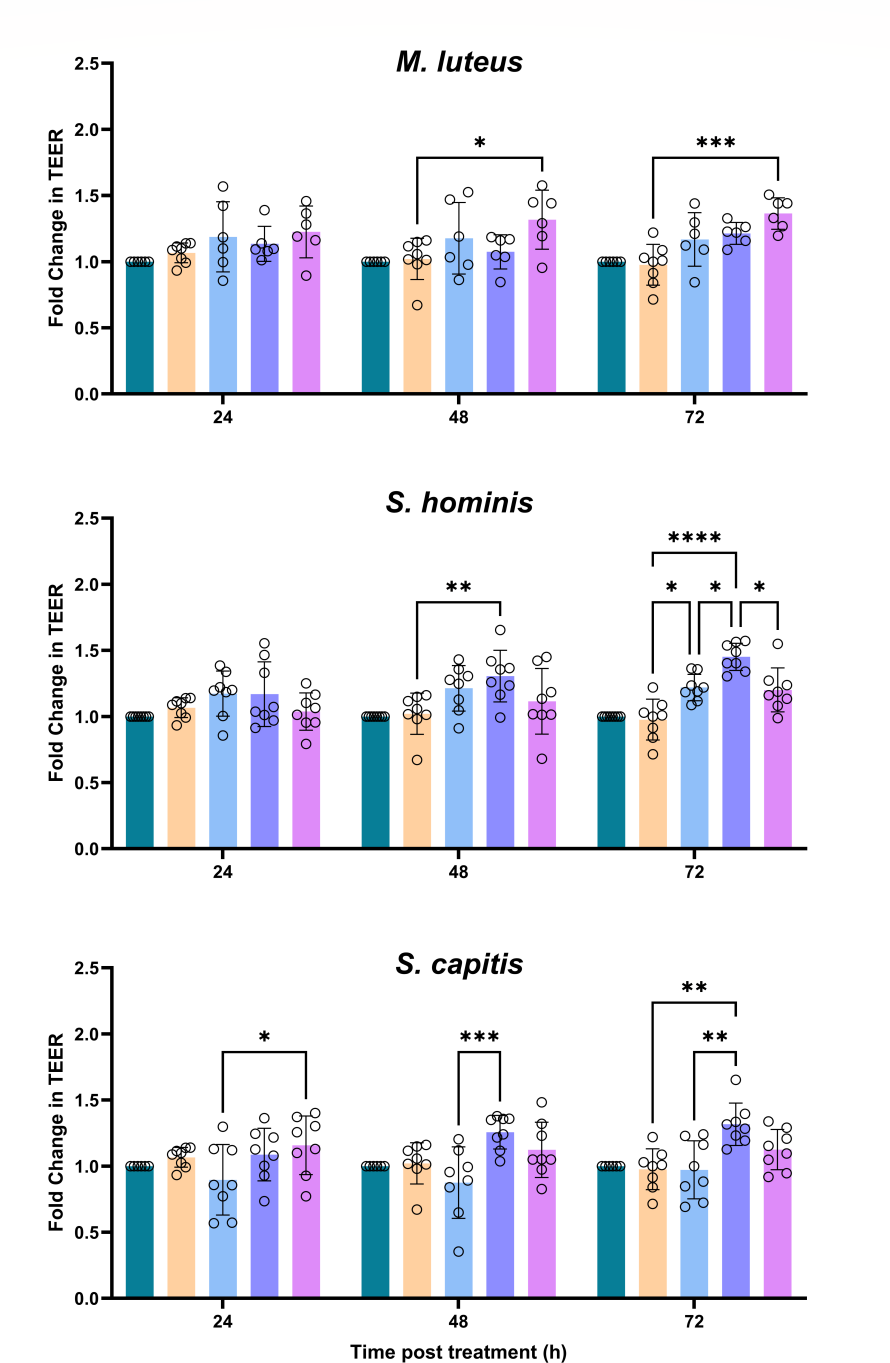
Fold change in trans-epithelial electrical resistance (TEER) of NHEKs after treatment with CFSNs from irradiated and unirradiated bacteria for 72 h. Control: no treatment, RPMI: vessel control. CFSNs (0, 37.5, and 150) are shown by the dose of SSR in mJ. Biological *n* = 6–8. One-way ANOVA with a Tukey's *post hoc*. *, *P* < 0.05; **, *P* < 0.01; ***, P < 0.001; ****, *P* < 0.0001.

Finally, as tryptophan derivatives are known to increase epithelial barrier integrity via AhR signaling, we assessed how SSR-induced changes in metabolite production affect the barrier function of primary human keratinocytes using TEER (Fig. 6).

When unirradiated, only *S. hominis* CFSNs significantly increased TEER, and for many organisms, irradiation did not change the impact of their metabolome on TEER. However, irradiation of *M. luteus*, *S. hominis*, and *S. capitis* resulted in CFSNs that enhanced TEER. After 72 h, the CFSN from irradiated *M. luteus* produced a 24% increase in TEER compared to the RPMI control after irradiation with 150 mJ (*P* = 0.0004), which was 14% greater than the 0 mJ CFSNs (observed increase only). *S. hominis* increased TEER at all doses of SSR compared to the RPMI control (0 mJ: *P* = 0.0271, 37.5 mJ: *P* < 0.0001, and 150 mJ: *P* = 0.0515), and this was augmented by 16% compared to the unirradiated control after irradiation with 37.5 mJ (*P* = 0.0369). *S. capitis* also increased TEER after irradiation with 37.5 mJ of SSR compared to the 0 mJ control (*P* = 0.0023). To confirm that these effects were AhR mediated, AhR activity was inhibited by the antagonist CH-223191 prior to treatment with the CFSNs; this inhibited all increases in TEER (Fig. S5).

## DISCUSSION

In this study, we demonstrated that skin commensals have distinct, organism-specific metabolomes, and that UVR significantly alters these, at least in part by altering gene expression. This can have significant downstream consequences on AhR signaling and, in some cases, barrier-modulating properties. To our knowledge, the effects of UVR on skin commensal metabolism have not previously been explored, and therefore, the downstream effects of this have also not been determined.

Although evidence in skin bacteria is limited, there are several studies that report the effects of UVR on microbial metabolism and gene expression. Kumar et al. (21) reported that environmentally isolated *Enterobacter cloacae* had an altered whole-cell protein profile after exposure to UV-B. Additionally, expression was significantly increased for several genes, primarily relating to the UV-stress response. Chen (22) reported that UVR inhibited bacterial amino acid and lipid metabolism in wastewater samples and altered gene expression of nitrogen metabolism genes. Another study examined the effects of UVB on marine bacteria and found that irradiation reduced the activity of lipase and leucine-aminopeptidase, potentially altering microbial access to lipids and peptides (23). As metabolism is primarily enzyme-driven, inactivity could inhibit the production of metabolites.

In this study, irradiation with 37.5 mJ SSR significantly upregulated *ipdC* (indole-3-pyruvate decarboxylase), *trpE* (anthranilate synthase component I), and *ALDH* (aldehyde dehydrogenase) in *S. hominis*. Functionally, *trpE* initiates *de novo* tryptophan biosynthesis from chorismate; *ipdC* converts indole-3-pyruvate to indole-3-acetaldehyde; and *ALDH* oxidizes this aldehyde to indole-3-acetic acid (24, 25). Given the breadth of genes contributing to tryptophan synthesis and downstream metabolism, as well as differential expression between species or even strains (5, 25), additional transcriptional changes likely underlie the observed metabolomic shifts. Nonetheless, our findings demonstrate that UVR can modulate key enzymes in this pathway, highlighting the need to explore other UVR-responsive steps in the tryptophan metabolism pathway.

High-dose UVR (150 mJ) generally reduced the abundance of metabolites for many organisms. This is likely due to reduced bacterial abundance as well as direct inhibition of microbial processes. Excess exposure of UVR to microbes is known to increase the production of reactive oxygen species (ROS) and damage DNA by the production of pyrimidine dimers and 6–4 photoproducts, which can stall RNA polymerases and trigger repair pathways (4, 26); this, in turn, can lead to downregulation of non‐essential metabolic genes (27). In plants, for example, UVB exposure has been shown to damage DNA and reduce transcription of genes in pathways not immediately involved in repair or protective pigmentation, shifting metabolism away from secondary metabolism under high UVR stress (28). Alongside transcriptional changes, UVR also directly affects enzyme activity through oxidative damage. UVR exposure produces ROS that can oxidize amino acid side chains, metal cofactors, or prosthetic groups in enzymes, disrupting their catalytic function (29, 30). For example, UVR has been shown to impair key antioxidant enzymes such as catalase and superoxide dismutase in algal and bacterial systems, decreasing their activity after UVB stress (31). Interestingly, increases in metabolism, particularly tryptophan metabolism, have not, to our knowledge, been reported.

Increased presence of indole-3-acetamide, tryptophol, indole-3-carboxaldehyde (IAld/I3A), and tryptamine correlated with increased AhR signaling. This has previously been reported for these metabolites (32, 33). IAld was found to be significantly decreased in atopic dermatitis, and topical application attenuated barrier defects by increasing AhR signaling (34). Tryptophol (indole-3-ethanol) and IAld have also been shown to regulate the gut barrier via AhR activation (35). Increased production of these metabolites following exposure to UVR could be one of the key ways the microbiome regulates the skin barrier’s response to UVR.

Although several organisms showed similar or reduced tryptophan-pathway metabolites after high-dose UVR, AhR activity often increased. For example, *S. hominis* at 150 mJ exhibited a diminished tryptophan metabolome yet increased AhR activation. One explanation for this is the ligand mix and differences in affinity and efficacy of the available metabolites, which have been previously reported (32). Our GC–MS data demonstrated that UVR can depress concentrations of potentially low-efficacy/partial agonists (including non-tryptophan metabolites that still occupy the receptor), reducing competitive occupancy and thereby permitting binding by higher-efficacy AhR agonists, which can drive greater transcriptional output even if their absolute levels are unchanged or reduced.

In some organisms (*M. luteus*, *S. hominis*, and *S. capitis*), increased AhR activation driven by UVR-induced changes in metabolome led to increased TEER, consistent with evidence that microbiome-derived AhR ligands can strengthen epithelial barriers in both gut and skin. Notably, however, elevated AhR activity did not universally translate into TEER changes, likely reflecting differences in the mixture, potency, and concentrations of metabolites, as distinct AhR ligands can elicit numerous responses. Future work should pinpoint the causal metabolites, as identifying the specific, high-efficacy microbial ligands could inform microbiome-based or metabolite supplementation strategies to enhance barrier recovery in disease or after sun exposure.

### Conclusion

Skin commensals generate tryptophan-derived metabolites that enhance barrier function through AhR signaling. This study demonstrates that UVR elevates expression of tryptophan-metabolism genes and shifts the microbial metabolome, most notably at lower UVR doses, toward increased presence of tryptophan metabolites, with corresponding increases in AhR activity and, in some cases, improved epithelial barrier integrity. This highlights one of the ways the skin microbiome potentially regulates the skin’s response to UVR, by shifting metabolite production, altering AhR signaling, and enhancing barrier integrity following photoexposure. Future studies should pinpoint the specific metabolites driving these effects and clarify the contribution of microbial metabolites in photo-exposed skin.

## Data Availability

The raw LC–MS and GC–MS metabolomics data generated in this study have been deposited in Zenodo and are available under DOI: https://doi.org/10.5281/zenodo.18349217. The remaining data are available upon request of the corresponding author.

## SUPPLEMENTAL MATERIAL

Supplemental figures aem.02385-25-s0001.docx Figures S1 to S5.

Supplemental tables aem.02385-25-s0002.docx Tables S1 and S2.

ASM does not own the copyrights to Supplemental Material that may be linked to, or accessed through, an article. The authors have granted ASM a non-exclusive, world-wide license to publish the Supplemental Material files. Please contact the corresponding author directly for reuse.
