# Supplementary material for: Ultraviolet radiation reshapes the metabolome of skin commensal bacteria, influencing AhR signaling and barrier function
Source: Appl Environ Microbiol. 2026 Mar 18;92(4):e02385-25. doi: 10.1128/aem.02385-25 (PMC13101469; doi:10.1128/aem.02385-25)
Supplement: Supplemental figures — Figures S1 to S5. [file aem.02385-25-s0001.docx]

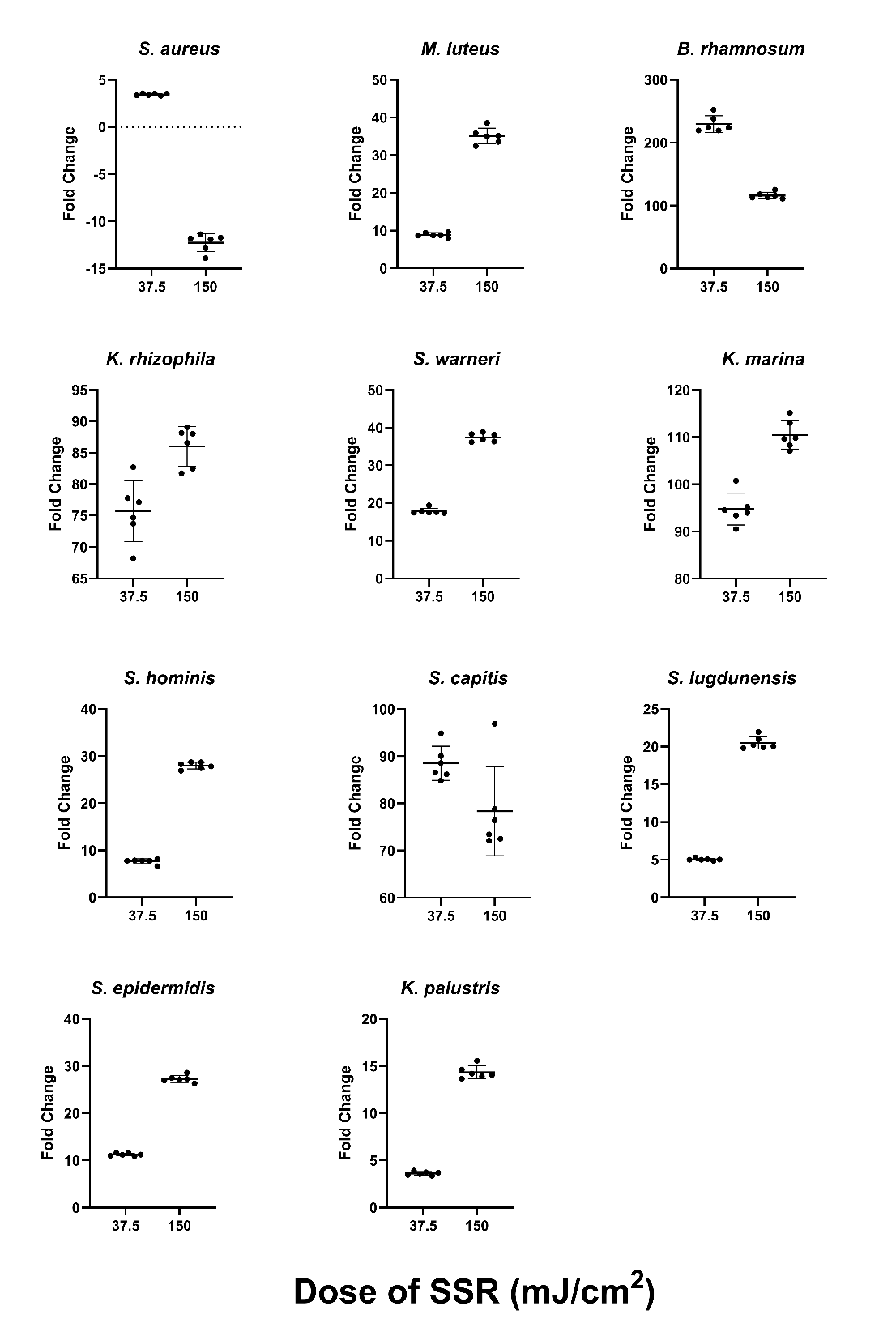


**Supplemental Figure 1.** Production of reactive oxygen species (ROS) by skin commensals following irradiation with 37.5 or 150mJ of SSR. Data is presented as mean fold change (+/- SD) compared to the unirradiated control. Biological n = 6.


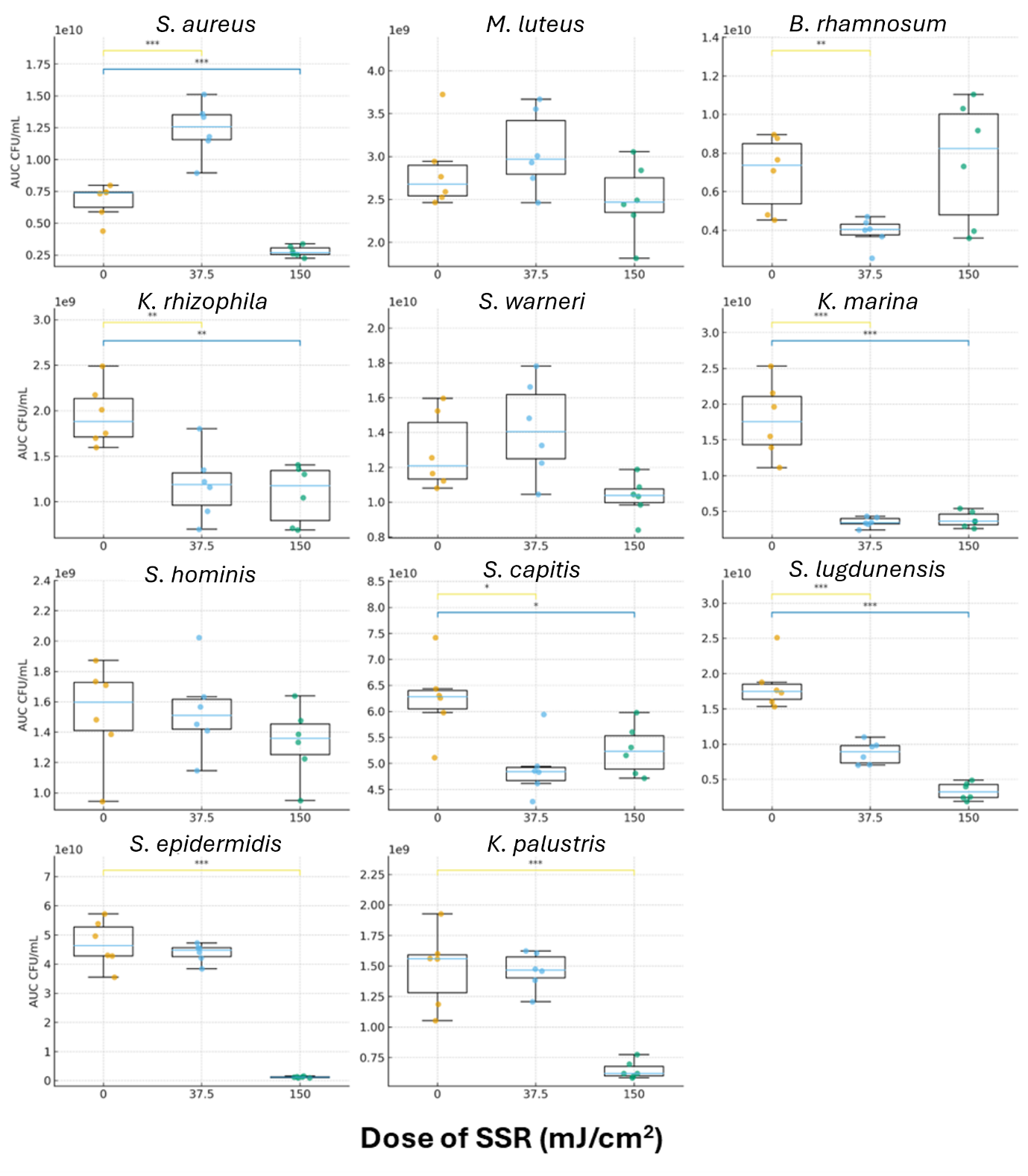


**Supplemental Figure 2.** Area under the curve of bacterial cell counts (AUC; CFU/mL) for each organism after irradiation with SSR. CFU counts were performed at 0, 24, 48, 72, 96 and 120h post irradiation. Significant differences relative to the unirradiated control were determined using one-way ANOVA followed by Holm-adjusted planned comparisons; *(P < 0.05), **(P < 0.01), ***(P < 0.001), ****(P < 0.0001).


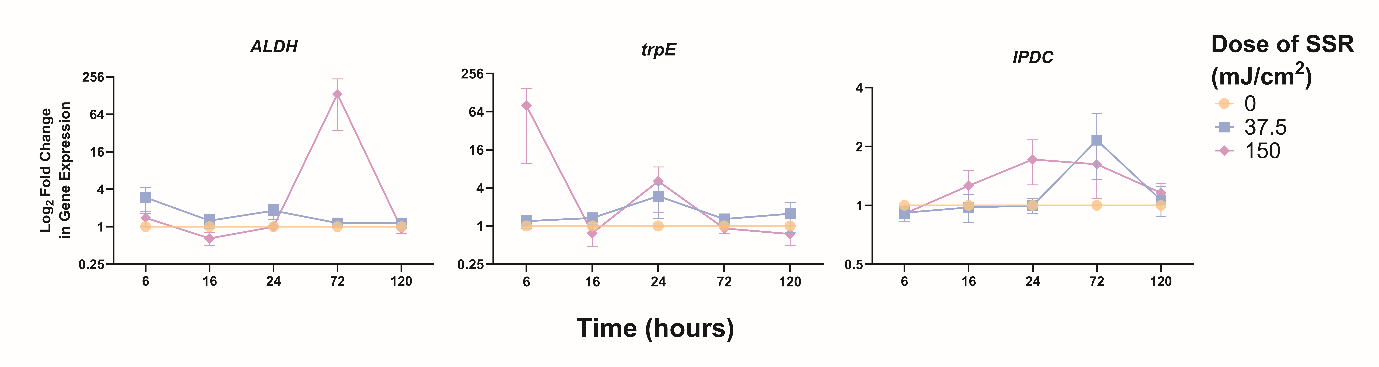


**Supplemental Figure 3.** Expression of tryptophan biosynthesis and metabolism genes following irradiation with SSR. Log_2_ fold-changes (2^-ΔΔCT^) are shown for a 120-h time course. Genes analysed include indole-3-pyruvate decarboxylase (*ipdC*), anthranilate synthase component 1 (*trpE*), and aldehyde dehydrogenase (*ALDH*). Data represent biological replicates (n = 4 - 5).


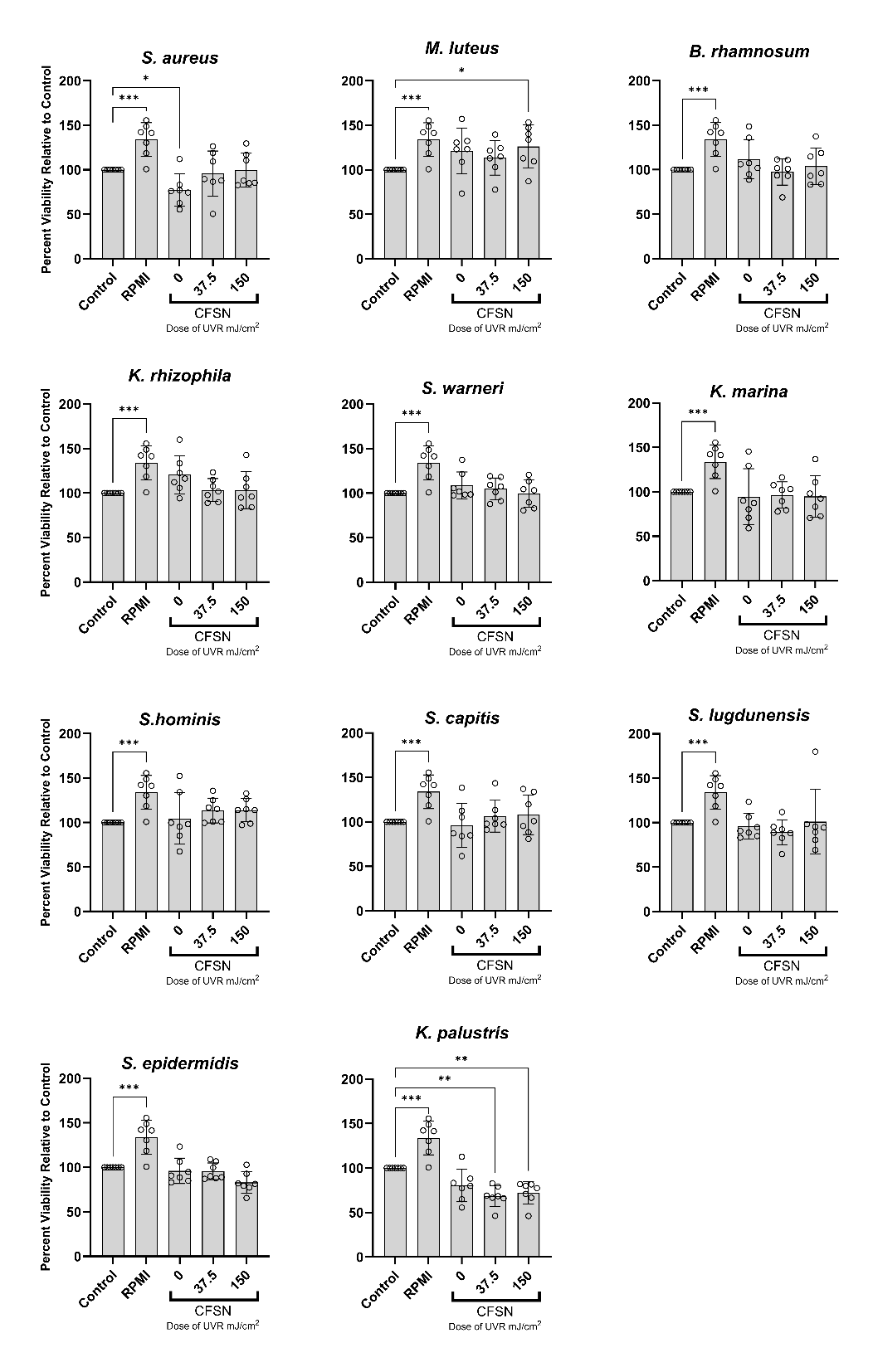


**Supplemental Figure 4.** Cell viability of NHEKs determined by MTT following 24h treatment with unirradiated and irradiated CFSNs. Biological n=7. Kruskal-Wallis test with a Dunn’s posthoc: *(*P* < 0.05), **(*P* < 0.01), ***(*P* < 0.001), ****(*P* < 0.0001).

**Supplemental Figure 2.** Fold change in transepithelial electrical resistance (TEER) of NHEKs treated for 72 h with CFSNs from irradiated vs. unirradiated bacteria in the presence of an AhR inhibitor. Biological n = 5; no significant differences detected.


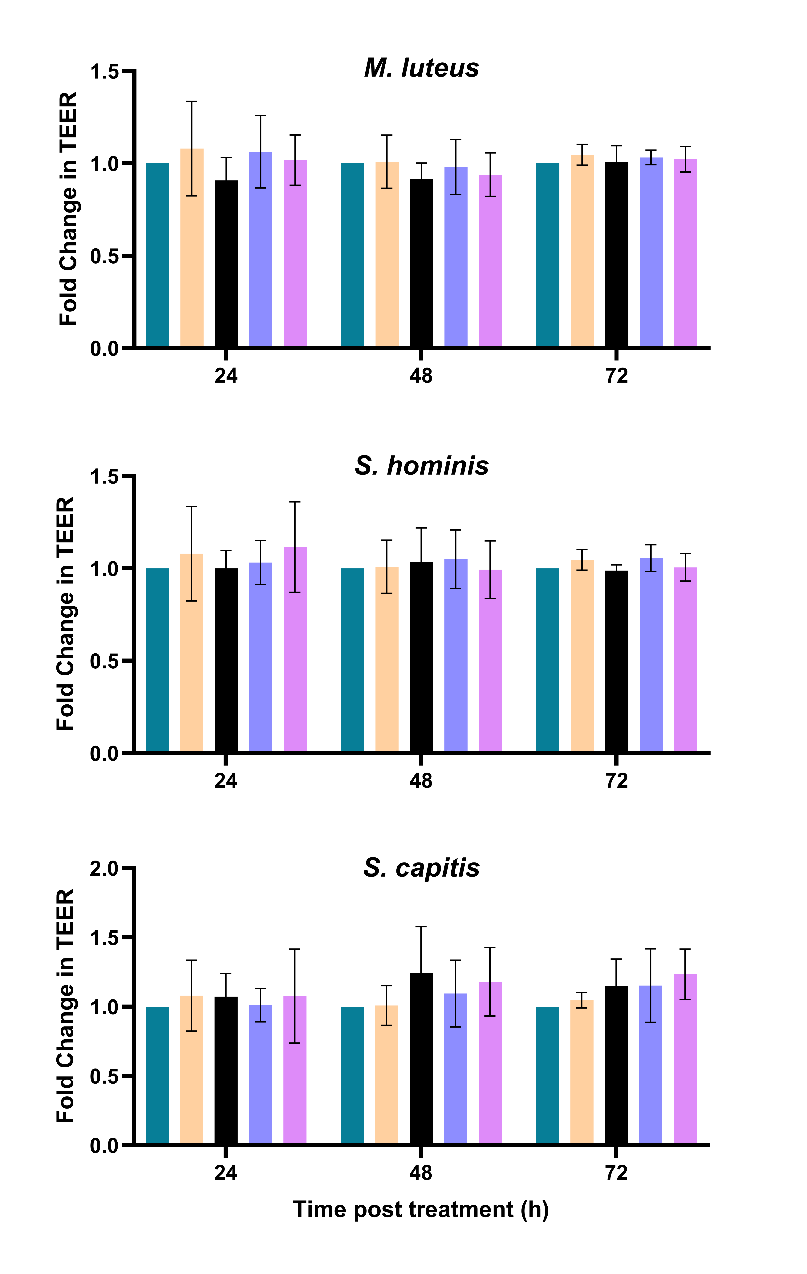


**Supplemental Figure 5.** Fold change in trans-epithelial electrical resistance (TEER) of NHEKs after treatment with CFSNs from irradiated and unirradiated bacteria for 72h in the presence of AhR antagonist. Control: no treatment, RPMI: vessel control. CFSNs (0, 37.5, 150) are shown by the dose of SSR in mJ. Biological n=5. One-way ANOVA with a Tukeys posthoc, no significant changes recorded.
