# Supplementary material for: Ultraviolet radiation reshapes the metabolome of skin commensal bacteria, influencing AhR signaling and barrier function
Source: Appl Environ Microbiol. 2026 Mar 18;92(4):e02385-25. doi: 10.1128/aem.02385-25 (PMC13101469; doi:10.1128/aem.02385-25)
Supplement: Supplemental tables — Tables S1 and S2. [file aem.02385-25-s0002.docx]

**Supplemental Table 1.** Identification of selected bacterial isolates based on 16S rRNA gene sequence analysis. Taxonomic assignments were determined by BLAST comparison of isolate 16S rRNA gene sequences against NCBI reference databases. Percent identity, query coverage, and the corresponding reference 16S rRNA gene accession numbers for the closest matching sequences are reported.

| **Closest BLAST match (16S rRNA)** | **Sequence identity (%)** | **Query coverage (%)** | **Reference sequence accession (NCBI)** | **Reference 16S rRNA length (bp)** | **BLAST query length (bp)** |
| --- | --- | --- | --- | --- | --- |
| *Staphylococcus aureus* strain S33 | 97.95% | 97% | NR_037007.2 | 1552 | 1043 |
| *Micrococcus luteus* strain NCTC 2665 | 97.15% | 100% | NR_075062.2 | 1525 | 1241 |
| *Brachybacterium rhamnosum* strain H-6S | 95.79% | 98% | NR_042109.1 | 1471 | 792 |
| *Kocuria rhizophila* strain TA68 | 97.00% | 96.55% | NR_026452.1 | 1471 | 1172 |
| *Staphylococcus warneri* strain AW 25 | 93.00% | 95.88% | NR_025922.1 | 1470 | 982 |
| *Kocuria marina* strain KMM 3905 | 99.00% | 98.33% | NR_025723.1 | 1440 | 954 |
| *Staphylococcus hominis* subsp. *novobiosepticus* strain GTC 1228 | 98.00% | 97.73% | NR_041323.1 | 1454 | 1065 |
| *Staphylococcus capitis* strain JCM 2420 | 99.00% | 98.79% | NR_113348.1 | 1473 | 1152 |
| *Staphylococcus lugdunensis* strain ATCC 43809 | 97.00% | 99.54% | NR_024668.1 | 1492 | 671 |
| *Staphylococcus epidermidis* strain NBRC 100911 | 98.00% | 97.22% | NR_113957.1 | 1476 | 1233 |
| *Kocuria palustris* strain Abk-6 | 99.00% | 98.25% | KF704371.1 | 1434 | 459 |

**Supplemental Table 2.** **Primer and probe sequences for custom TaqMan qPCR assays**

Table shows reference sequence information submitted to the ThermoFisher Scientific custom TaqMan® assay design service, together with the primer and probe sequences generated by ThermoFisher Scientific. ThermoFisher custom assay IDs are provided for assay identification and re-ordering.

| **Target** | **Target name** | **Reference sequence** | **ThermoFisher custom assay ID** | **Forward primer (5′–3′)** | **Reverse primer (5′–3′)** | **Probe (5′–3′)** |
| --- | --- | --- | --- | --- | --- | --- |
| ipdC | Indole-3-pyruvate decarboxylase | *Staphylococcus hominis* FDAARGOS_575 genome (RefSeq: **CP134693.1**) | AP2XKMK | CAATGCAATTTCTGTATCAATCGTATCGT | CCACTGCAGGCTTTTCATATCAAT | CCACGTCGTTAATATC |
| ALDH | Aldehyde dehydrogenase | *Staphylococcus hominis* FDAARGOS_575 genome (RefSeq: **CP033732.1**) | APYMXGR | CTGGTTCAACAAGTGTAGGTTATGGT | ACCGCCAAGTTCTAACGTAGTTG | TCAGCGCCTGCTTTAG |
| trpE | Anthranilate synthase component 1 | *Staphylococcus hominis* FDAARGOS_575 gene (locus tag: **EGX58_RS02720**) | APXG3WU | GGCACTATCTCAAATTAAAGCTTATACAGATGA | ATCGCCTTCTTTTATAAGTTCTTTCAATTGTTTT | ACGCCTTCAAATATCA |
| rpoB | DNA-directed RNA polymerase subunit β | *Staphylococcus hominis* FDAARGOS_575 gene (NCBI Gene ID: **58105916**) | AP33E7H | CTGCTGATGAAGAAGATAGCTATGTAGTT | CACTTCATCATCTAGGAAACGACCAT | CACAAGCCAATTCTCG |
